# Impact of Zr-Doped Bi_2_O_3_ Radiopacifier by Spray Pyrolysis on Mineral Trioxide Aggregate

**DOI:** 10.3390/ma14020453

**Published:** 2021-01-19

**Authors:** Tzu-Yu Peng, May-Show Chen, Ya-Yi Chen, Yao-Jui Chen, Chin-Yi Chen, Alex Fang, Bo-Jiun Shao, Min-Hua Chen, Chung-Kwei Lin

**Affiliations:** 1 Department of Anatomy and Functional Restorations, Graduate School of Biomedical and Health Sciences, Hiroshima University, Hiroshima 734-8553, Japan; typ@mail.cmu.edu.tw; 2 Research Center of Digital Oral Science and Technology, College of Oral Medicine, Taipei Medical University, Taipei 110, Taiwan; maychen@tmu.edu.tw (M.-S.C.); t10990@ms.sltung.com.tw (Y.-Y.C.); t12090@ms.sltung.com.tw (Y.-J.C.); chencyi@fcu.edu.tw (C.-Y.C.); 3 School of Dentistry, College of Dentistry, China Medical University, Taichung 404, Taiwan; 4 Division of Prosthodontics, Department of Dentistry, Taipei Medical University Hospital, Taipei 110, Taiwan; 5 School of Dentistry, College of Oral Medicine, Taipei Medical University, Taipei 110, Taiwan; 6 Department of Dentistry, Tung’s Taichung MetroHarbor Hospital, Taichung 433, Taiwan; 7 Department of Engineering Technology and Industrial Distribution, Texas A&M University, College Station, TX 77843, USA; gpafang@tamu.edu; 8 Department of Materials Science and Engineering, Feng Chia University, Taichung 407, Taiwan; gl1irmp529981@gmail.com; 9 Department of Biomedical Engineering, Chung Yuan Christian University, Taoyuan City 320, Taiwan; 10 Institute of Biomedical Engineering and Nanomedicine, National Health Research Institutes, Miaoli County 350, Taiwan; 11 School of Dental Technology, College of Oral Medicine, Taipei Medical University, Taipei 110, Taiwan; 12 Additive Manufacturing Center for Mass Customization Production, National Taipei University of Technology, Taipei 10608, Taiwan

**Keywords:** mineral trioxide aggregate, radiopacifier, spray pyrolysis, zirconium-doped, bismuth oxide

## Abstract

Mineral trioxide aggregates (MTA) have been developed as a dental root repair material for a range of endodontics procedures. They contain a small amount of bismuth oxide (Bi_2_O_3_) as a radiopacifier to differentiate adjacent bone tissue on radiographs for endodontic surgery. However, the addition of Bi_2_O_3_ to MTA will increase porosity and lead to the deterioration of MTA’s mechanical properties. Besides, Bi_2_O_3_ can also increase the setting time of MTA. To improve upon the undesirable effects caused by Bi_2_O_3_ additives, we used zirconium ions (Zr) to substitute the bismuth ions (Bi) in the Bi_2_O_3_ compound. Here we demonstrate a new composition of Zr-doped Bi_2_O_3_ using spray pyrolysis, a technique for producing fine solid particles. The results showed that Zr ions were doped into the Bi_2_O_3_ compound, resulting in the phase of Bi_7.38_Zr_0.62_O_12.31_. The results of materials analysis showed Bi_2_O_3_ with 15 mol % of Zr doping increased its radiopacity (5.16 ± 0.2 mm Al) and mechanical strength, compared to Bi_2_O_3_ and other ratios of Zr-doped Bi_2_O_3_. To our knowledge, this is the first study of fabrication and analysis of Zr-doped Bi_2_O_3_ radiopacifiers through the spray pyrolysis procedure. The study reveals that spray pyrolysis can be a new technique for preparing Zr-doped Bi_2_O_3_ radiopacifiers for future dental applications.

## 1. Introduction

Mineral trioxide aggregates (MTA) have been used as a root repair material for a range of endodontics procedures [1]. The main crystalline phases of MTA consist of dicalcium silicate (CaSiO_4_), tricalcium silicate (Ca_3_SiO_5_), and tricalcium aluminate (Ca_3_Al_2_O_6_), that is, a chemical similarity to Portland cement (PC) [2,3]. Additionally, it contains 20 wt% of radiopacifier to enhance its imaging contrast from adjacent bone tissue on radiographs for endodontic surgery [4]. Many radiopacifiers, such as barium sulfate (BaSO_4_), iodoform (CHI_3_), and bismuth oxide (Bi_2_O_3_), have been proposed as additives to MTA [5]. Among these radiopacifiers, Bi_2_O_3_ has the highest radiopacity value (approximately 5 mm Al) and is the most commonly used in MTA. Although Bismuth-based compounds have been often used in cosmetic and medical applications, there are many concerns about their intrinsic toxicity. They have been reported to induce oxidative stress in the blood [6]. Loman et al. [7] found that Bi_2_O_3_ particles caused genotoxic activity and raised the Allium cepa root meristematic cells’ mitotic index. Besides, the addition of Bi_2_O_3_ in MTA will also cause its porosity to increase from 15% to 31%, leading to deterioration in mechanical properties [8].

Recently, commercial products such as NeoMTA Plus (Avalon Biomed Inc., Houston, TX, USA) and MTA Repair HP (Angelus Indústria de Produtos Odotontológicos S/A, Londrina, Brazil), which are based on tricalcium silicates, have been introduced [9]. NeoMTA Plus and MTA Repair HP are incorporated with tantalum oxide and calcium tungstate, respectively, as a radiopacifier instead of bismuth oxide. Though the two products are not notable different from the traditional MTA, post-marketing surveillance in public is still underway. Therefore, we believe the conventional MTA still needs to be further explored. To reduce the effects of Bi_2_O_3_ additives, we used zirconium ions (Zr) to substitute part of the bismuth ions in Bi_2_O_3_. Numerous studies have shown that the physical and chemical properties of Bi_2_O_3_ can be regulated by different metallic ion doping. For instance, when bismuth ions (Bi) in Bi_2_O_3_ is replaced by ions such as Ni^2+^ and La^3+^, this Bi_2_O_3_ compound could become a useful agent in improving the photoresponse of the material [10,11]. Bi_2_O_3_ with Ta^5+^ doping can improve chemical stability and has improved materials to be more environmentally friendly [12]. These findings demonstrate that Bi_2_O_3_ can be a host material for metallic ions substitution, which converts Bi_2_O_3_ into a material with desired properties. Zr has been found as a practical strengthening element. Many researchers used Zr as a dopant ion to increase the mechanical properties in materials such as hydroxyapatite and titanium alloy [13,14]. Moreover, Djordje et al. [15] suggest that zirconium dioxide could be an alternative radiopacifier to replace Bi_2_O_3_ in MTA without influencing its physical properties. 

Another concern of MTA is its long setting time. Liu et al. [16] reported that the setting time’s efficiency could be strongly affected by the constituent particles’ shapes. Thus, to address a long setting time, developing much smaller and more homogeneous radiopacifiers was required for unmet clinical needs. The Zr-doped Bi_2_O_3_ radiopacifier can be easily prepared by sol-gel procedure, but this will result in the irregular particle shapes and random particle sizes being composed [17,18]. We addressed this issue in the previous study by preparing bismuth/zirconium oxide composite powder through high energy ball milling [19]. We further demonstrated a proof of concept as a new Zr-doped Bi_2_O_3_ radiopacifier using spray pyrolysis. This technique has been proved to synthesize the powders of a narrow particle size distribution [20]. Through spray pyrolysis, materials can be synthesized with a smaller and more homogenous spherical shape than those of the sol-gel method. 

Furthermore, the particles can be quickly produced through a spray pyrolysis technique in one step. Whereas the sol-gel process typically requires several steps and may increase the production cost [21]. We are the first to synthesize the radiopacifiers using the spray pyrolysis procedure to the best of our knowledge. In the study, 20 wt% of Zr-doped Bi_2_O_3_ prepared by spray pyrolysis was mixed in PC (80 wt%) and tested for the radiopacity, mechanical strength, and setting time.

## 2. Materials and Methods

### 2.1. Synthesis of Zr-Doped Bi_2_O_3_ Particles

In the study, we used spray pyrolysis to prepare Bi_2-x_ZrxO_3+x/2_ composite powder to serve as the radiopacifier within MTA. Preparation of Bi_2-x_Zr_x_O_3+x/2_ particles with different molar ratios of Zr doping was conducted by the hydrolysis and condensation reactions under the procedure of spray pyrolysis. All chemicals were of analytical grade and used as received without further purification. First, bismuth nitrate pentahydrate (Bi(NO_3_)_3_∙5H_2_O) and glacial acetic acid (CH_3_COOH) were mixed under mild stirring for 30 min. Then, zirconyl nitrate hydrate (ZrO(NO_3_)_2_∙H_2_O) with various ratios was added to the mixing solutions and was mildly stirred for another 60 min. After that, an ultrasonic humidifier (KT-100A, King Ultrasonics Co., Ltd., Taiwan) with a frequency of 1.65 MHz was applied to the mixed solution to generate droplets. The generated droplets were then rapidly heated in the furnace up to 750 °C. After cooling down to room temperature, the obtained dried particles were prepared for analysis. Samples were prepared from different molar ratios of Zr doping, which are representatives of Zr (10 mol %): Bi_2_O_3_; Zr (15 mol %): Bi_2_O_3_; and Zr (20 mol %): Bi_2_O_3_. MTA was prepared by mixing the powers of PC (80 wt%) and radiopacifiers (Bi_2_O_3_ or Bi_2-x_Zr_x_O_3+x/2_, 20 wt%) at a powder/liquid ratio of 3:1.

### 2.2. Characterization

The morphologies of particles were evaluated using field emission scanning electron microscopy (SEM; JSM-6700F, JEOL, Tokyo, Japan). High resolution of microstructure was observed by dropping samples onto a copper grid using transmission electron microscopy (TEM; JEOL-2100F, JEOL, Tokyo, Japan), operated at an accelerating voltage of 200 kV. Powder X-ray diffraction (XRD: MacScience, Yokohama, Japan) was utilized to identify the crystalline phase composition using Cu Kα radiation with the potential at 30 kV and the current at 20 mA. Thermogravimetric and differential thermal analysis (TGA/DTA; SDT2960, TA Instrument, New Castle, DE, USA) was employed to investigate particles’ decomposition behavior with increasing temperature at 30 °C min^−1^.

The powders’ radiopacity was determined using a dental X-ray system (VX-65, Vatech Tech, Gyeonggido, Korea). The X-ray source was set at 62 kV and 10 mA with 30 cm µfocus-film distance, according to the guideline of ISO:6876-2012.

The mechanical properties of materials were evaluated by the diametral tensile strength, which is a property described by the American National Standards Institute/ Amercican Dental Association (ANSI/ADA) Specification 27 for characterizing dental composite restoratives [22]. The procedure was performed following ISO9917-1 standards. The Zr (15 mol %): Bi_2_O_3_ mixed with PC was poured into a mold (5 mm in diameter and 6 mm in height) and was measured using a universal testing machine (Lloyd LR MK1; Lloyd Instruments Ltd, West Sussex, UK). Before the diametral tensile strength study, we first confirmed that the samples were prepared well and consistently. The tests were recorded with a 500 N load cell and crosshead rate of 1 mm/s until the sample failed.

Setting time was evaluated according to ISO 9917-1:2007(E). The mixing of PC and radiopacfiers was compacted into glass molds with a diameter of 5 mm and 6 mm in height. Testing was performed using a modified Vicat apparatus (ASTM 187-19; Torontech Inc., Markham, Canada), which consisted of a weighted needle. The samples were tested by perpendicularly loading a weighted needle onto the samples’ plane. The initial setting time was calculated when a depth of press of 1 mm was reached; the final setting time was determined when the surface with no noticeable indentation appeared. MTA without radiopacifier (100 wt% of PC) was used as a control. The measurement was tested on twelve samples.

All of the values above were presented as mean ± standard error of the mean of at least five repeats. Statistical analysis was performed using the analysis student’s paired *t*-test. Values of * *p* < 0.05 were considered statistically significant. The calculations were performed using SPSS version 18.0 software (IBM Corporation, NY, USA).

## 3. Results and Discussion

### 3.1. Characteristics of Bi_2_O_3_

**Figure 1:**
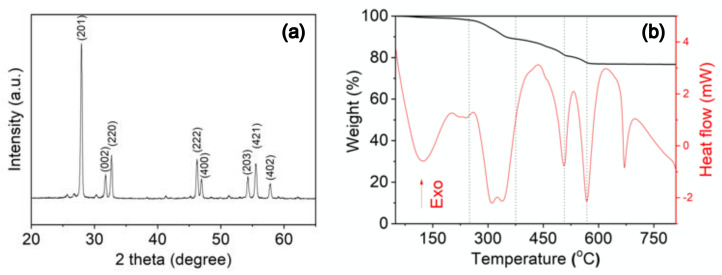
Characterization of Bi_2_O_3_ particles. (**a**) XRD pattern; and (**b**) TGA/ DTA analysis.

**Figure 2:**
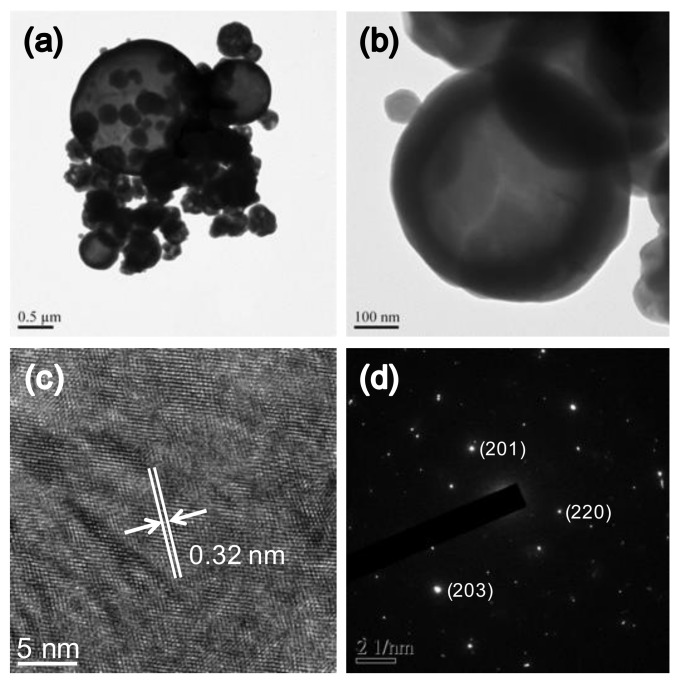
Characterization of Bi_2_O_3_ particles. (**a**,**b**) TEM images; (**c**) crystal lattice planes; and (**d**) the diffraction pattern.

The study shows that the Bi_2_O_3_ radiopacifier can be synthesized through spray pyrolysis, followed by an annealing temperature at 750 °C. XRD suggested that the material was β-Bi_2_O_3_ crystal structure (JCPDS standard card no. 27-0050) (Figure 1a). In TGA/DTA analysis (Figure 1b), the exothermal peak was observed when the temperature reached up to 670 °C due to the phase transition from the monoclinic α-phase to the δ-phase [23,24]. However, when the material returned to room temperature, the structure transformed into the intermediate metastable tetragonal (β-Bi_2_O_3_) phase [23,25]. Other attendant weight loss (33%) within 250 °C represented the loss of water and organic species (nitrate, acetate, oxyhydroxide, and bismuth hydroxide) (Figure 1b). TEM images revealed that particles were almost spherical (Figure 2a,b). The materials had a small agglomeration, and the large particles (around 2 µm) had a few small particles (0.5 µm) on them. Within a single particle, lattice space value was measured to be 0.32 nm, corresponding to the d-space of the (201) plane in the β-structure of Bi_2_O_3_ crystal (JCPDS standard card no. 27-0050) (Figure 2c,d). As illustrated, the importance of these findings suggests that the Bi_2_O_3_ radiopacifier can be synthesized through the spray pyrolysis process.

### 3.2. Characteristics of Zr-Doped Bi_2_O_3_

**Figure 3:**
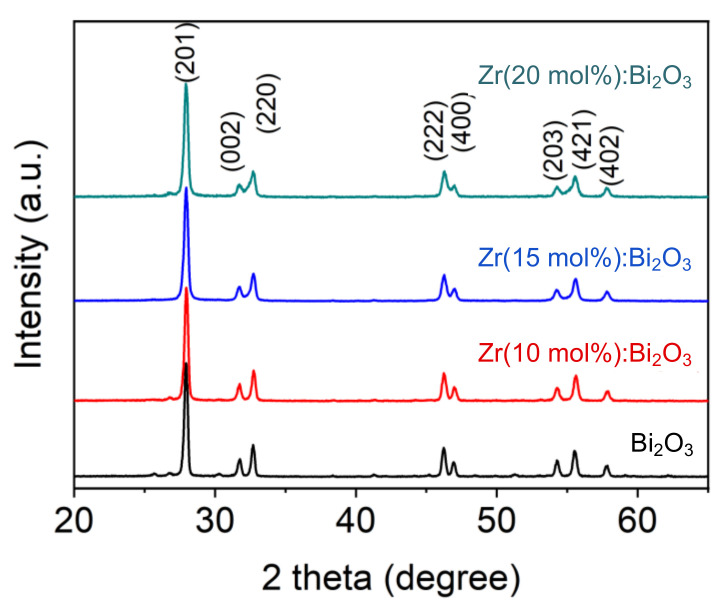
XRD patterns of Bi_2_O_3_; Zr (10 mol %): Bi_2_O_3_; Zr (15 mol %): Bi_2_O_3_; and Zr (20 mol %): Bi_2_O_3_.

Because of the encouraging results from the Bi_2_O_3_, we further prepared the Zr-doped Bi_2_O_3_ according to a similar procedure of preparing Bi_2_O_3_. Bi_2_O_3_ doped with Zr in different molar ratios (10, 15, 20 mol %) were evaluated. The peaks of XRD patterns were consistent with the (201), (002), (220), (222), (400), (203), (421) and (402) reflection, revealing the formation well indexed to β-structure of Bi_7.38_Zr_0.62_O_12.31_ (JCPDS standard card no. 43-0445) (Figure 3). As illustrated, no additional foreign peaks corresponding to the zirconia phase were observed, suggesting that the Zr was fully encapsulated or doped in Bi_2_O_3_ [26,27]. However, mild changes in peak position, intensity, and broadening were detected from the XRD spectrum. The lower intensity and a slight shift to larger angles with the increasing Zr adding are attributed to the lattice parameters’ variation resulting from Zr’s doping in Bi-O lattice [26]. The broadening peaks are reasonably due to the substitution of larger cations (Bi^3+^, 117 pm) by smaller cations (Zr^4+^, 86 pm), resulting in the inhibition of the crystals growth [28].

**Figure 4:**
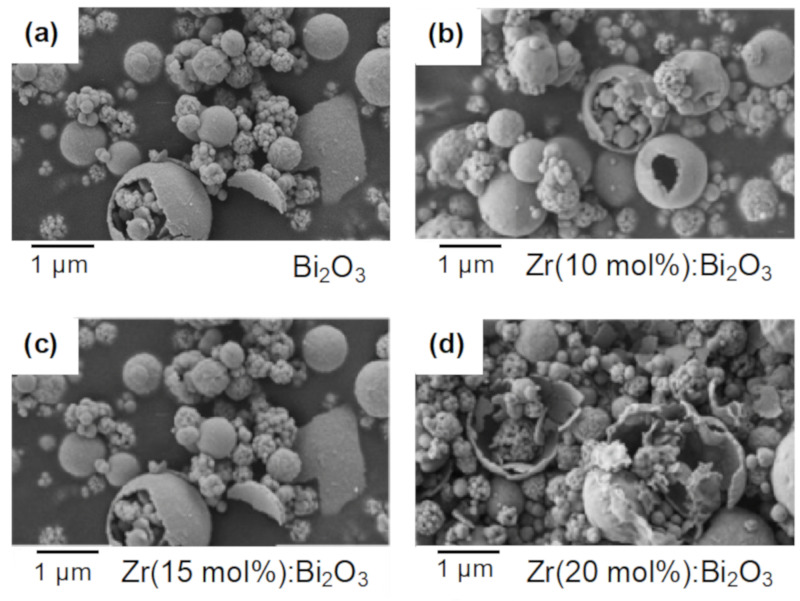
SEM images of (**a**) Bi_2_O_3_; (**b**) Zr (10 mol %): Bi_2_O_3_; (**c**) Zr (15 mol %): Bi_2_O_3_; and (**d**) Zr (20 mol %): Bi_2_O_3_.

**Figure 5:**
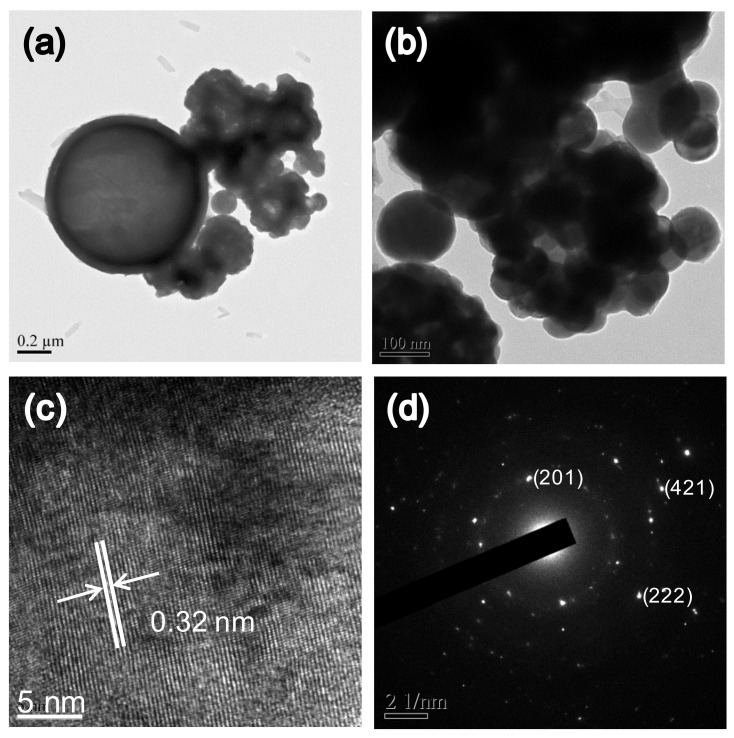
Characterization of Zr (15 mol %): Bi_2_O_3_ particles. (**a**,**b**) TEM images; (**c**) crystal lattice planes; and (**d**) the diffraction pattern.

The morphology and size distribution were similar whether or not Bi_2_O_3_ was doped with Zr. SEM micrographs revealed that Bi_7.38_Zr_0.62_O_12.31_ were also synthesized almost spherically (Figure 4). The materials had a small agglomeration with large particles (around 2 µm) and small particles (0.5 µm). Similar results were obtained with TEM images, and no unobvious second phases were shown on the particle surface (15 mol % of Zr doping is representative) (Figure 5). As known from the literature, Bi_7.38_Zr_0.62_O_12.31_ particles were formed by self-recrystallization and were the aggregative assemblies of Bi and Zr precursors through spray pyrolysis [23]. Compared with a sol-gel method, materials can be synthesized with a smaller and more homogenous spherical shape by using spray pyrolysis [18].

### 3.3. The Effect of Zr-Doped Bi_2_O_3_

**Figure 6:**
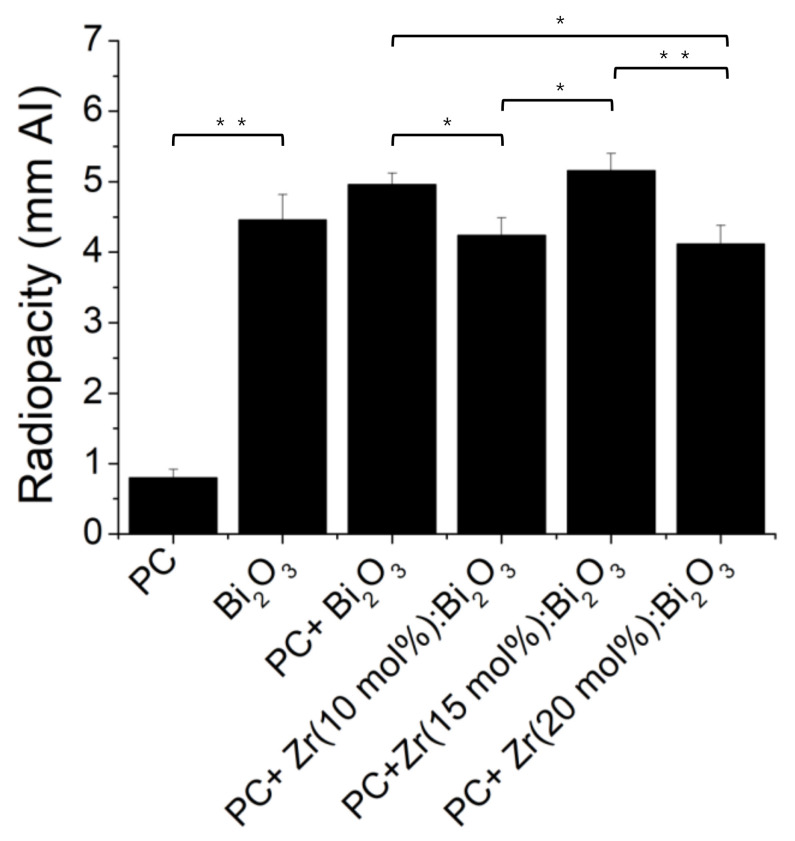
The radiopacity of Portland cement (PC) mixed with different Zr-doping ratios of Bi_2_O_3_ particles. (*n* = 5, * *p* < 0.05, ** *p* < 0.01).

To understand whether spray pyrolysis-derived Zr-doped Bi_2_O_3_ is suitable as a radiopacifier, we further analyzed its properties when mixed with PC, including the radiopacity, mechanical strength, and setting time. The purpose of adding radiopacifiers in MTA is to attenuate the X-ray intensity and because it can be used to distinguish between the tissue and MTA. According to ISO 6876/2001 standard, the minimum radiopacity value for root canal sealing materials should more than 3 mm Al. [29]. Here we assessed the radiopacity of spray pyrolysis-derived Bi_2_O_3_ and Zr-doped Bi_2_O_3_ under a dental X-ray system. The results showed that PC had low radiopacity; however, when PC was mixed with radiopacifiers (Bi_2_O_3_ and/or Zr-doped Bi_2_O_3_), all samples’ radiopacity was increased and higher than 3 mm Al, indicating that these spray pyrolysis-derived materials are suitable as a radiopacifier in dental applications (Figure 6). In these materials, Bi_2_O_3_ with 15 mol % of Zr doping had higher radiopacity under the X-ray excitation (5.16 ± 0.24 mm Al) compared with other ratios of Zr doping (*p* < 0.05), but there was no significant difference between Zr (15 mol %): Bi_2_O_3_ and Bi_2_O_3_.

**Figure 7:**
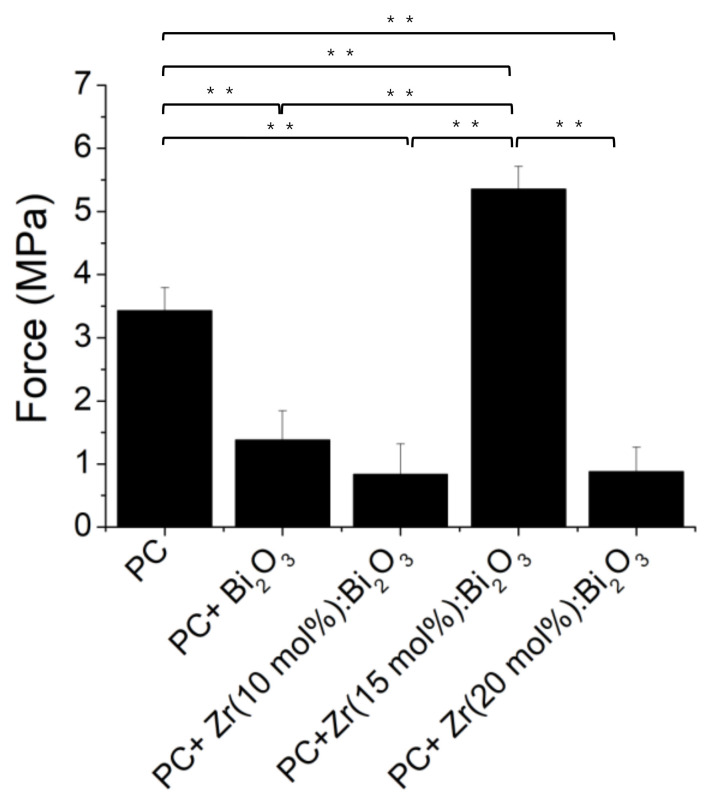
Diametral tensile strengths of PC mixed with different Zr-doping ratios of Bi_2_O_3_ particles. (*n* = 5, ** *p* < 0.01).

**Figure 8:**
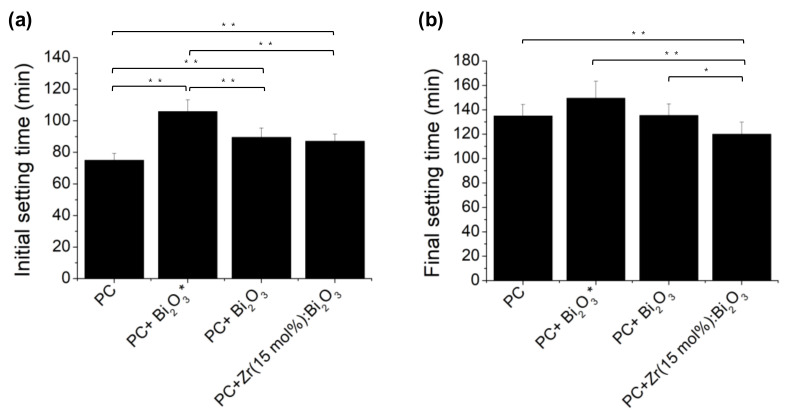
(**a**) Initial setting time; and (**b**) final setting time of PC mixed with spray pyrolysis-derived of Bi_2_O_3_ and Zr-doped Bi_2_O_3_ particles. Bi_2_O_3_* represents the sample from commercial sol-gel derived Bi_2_O_3_ powder. (*n* = 12, * *p* < 0.05, ** *p* < 0.01).

When we further assessed these materials to test their mechanical strength, PC mixed with 15 mol % of Zr-doped Bi_2_O_3_ also showed a higher mechanical strength than Bi_2_O_3_ and other Zr-doped ratios of Bi_2_O_3_ with PC (*p* < 0.01) (Figure 7). Although the reason for the higher mechanical strength of Zr (15 mol %): Bi_2_O_3_ is still unclear, it is most likely due to its microstructure of the crystalline phase [14]. According to Chen et al. [14], charge compensation is considered to induce defects and non-stoichiometry in the Bi_2_O_3_ lattice as tetravalent Zr substitution for trivalent Bi site. Regarding the Orowan mechanism, these defects will increase the resistance to lattice dislocation movement and enhance the diametral tensile strength of materials. To provide a proof of concept for using spray pyrolysis to produce a radiopacifier, the setting times of Bi_2_O_3_ with 15 mol % of Zr doping are illustrated as follows (Figure 8).

Although Zr-doped Bi_2_O_3_ radiopacifier can be easily prepared by sol-gel procedure, spray pyrolysis made Bi_2_O_3_ have a smaller and more homogenous spherical shape than current commercially used MTA powders [17,18]. According to S. Demirci et al. [21], inorganic samples derived from spray pyrolysis are better than those of the sol-gel method due to their physical properties, including specificity surface area and average particle size. Their study found the degradation efficiencies of spray pyrolysis- and sol-gel-derived nanoparticles were 94% and 90%, respectively. The increased degradation efficiency of spray pyrolysis can arise from the smaller particle size, enhancing the surface area-to-volume ratio of the catalysts, thereby increasing the number of reactive sites. According to the same theory, if we apply spray pyrolysis to prepare radiopacifiers, we also have the opportunity to shorten the setting time due to the faster hydration rate of calcium-silicate-hydrate gel reaction [20]. Our findings are similar to the suspicion that the initial setting times of PC mixed with spray pyrolysis-derived Bi_2_O_3_ were only about 90 min, while those combined with commercial sol-gel-derived Bi_2_O_3_ powder are more than 105 min (Figure 8a). The final setting time for the PC mixed with Zr (15 mol %): Bi_2_O_3_ appears to be shorter than those combined with Bi_2_O_3_ (*p* < 0.05) (Figure 8b).

Adding ZrO_2_ to replace Bi_2_O_3_ in MTA yields many advantages. However, as Bi_2_O_3_ is wholly replaced with ZrO_2_ in MTA, its radiopacity is decreased by about half [15]. Based on our past research findings [19], we added ZrO_2_ to Bi_2_O_3_ by ball milling method to form the phase of Bi_7.38_Zr_0.62_O_12.31_. Its radiopacity was decreased as the addition of ZrO_2_ due to the increasing amount of Zr with a relatively low radiodensity than Bi. Thus, we further demonstrate a proof of concept of a new Zr-doped Bi_2_O_3_ radiopacifier using spray pyrolysis. Through spray pyrolysis, materials can be synthesized with a smaller and more homogenous spherical shape than those of the sol-gel method. This study is the first to synthesize radiopacifiers using the spray pyrolysis procedure to the best of our knowledge. These results suggest that Zr (15 mol %): Bi_2_O_3_ synthesized by spray pyrolysis could be a new radiopacifier for future dental applications.

## 4. Conclusions

Here, we demonstrated a proof-of-concept radiopacifier using the spray pyrolysis technique. According to our preliminary finding, Bi_2_O_3_ and Zr-doped Bi_2_O_3_ compounds could be synthesized almost spherically. The particles’ size was a little agglomeration with the larger particles (around 2 µm) with a small number of small particles (0.5 µm) on their surface. Compared with the sol-gel process, radiopacifiers synthesized by spray pyrolysis had a shortened setting time. Additionally, we showed that Bi_2_O_3_ with 15 mol % of Zr doping showed the higher radiopacity under the X-ray excitation (5.16 ± 0.24 mm Al) and had substantially increased mechanical strength, compared to Bi_2_O_3_ and other ratios of Zr-doped Bi_2_O_3_ mixed with PC. The results further support Zr-doped Bi_2_O_3_ through spray pyrolysis as a new radiopacifier for future dental filling and pulp-capping applications. What needs to be investigated in the future are the effects of Zr when it is associated with PC and the impact of tooth discoloration.
